# Supplementary material for: Whole Locus Sequencing Identifies a Prevalent Founder Deep Intronic RPGRIP1 Pathologic Variant in the French Leber Congenital Amaurosis Cohort
Source: Genes (Basel). 2021 Feb 18;12(2):287. doi: 10.3390/genes12020287 (PMC7922592; doi:10.3390/genes12020287)

Supplementary Table S1 : LCA/EOSRD SureSelect panel.
A custom panel was designed to include genes involved in LCA, EOSRD, differential diagnosis and ciliopathies and neurometabolic diseases which can manifest initially as an LCA or an EOSRD according to the OMIM database or the literature (at least one reported case). The target regions encompassed 468 kb, covered 966 exons, 1000 bases of 5' and 3' UTRs and 50bp spanning splice junctions of 55 genes.

name	transcript	chr	start	end	refseq	ccds	nb_exon	group	description
AHI1	ENST00000457866_6	6	1,36E+08	1,36E+08	NM_01765	CCDS47483	28	Leber	Abelson helper integration site 1
AIPL1	ENST00000381129_17	17	6327057	6338505	NM_01433	CCDS11075	6	Leber	aryl hydrocarbon receptor interacting protein-like 1
ALMS1	ENST00000264448_2	2	73612886	73837046	XM_00526	CCDS42697	23	Leber	Alstrom syndrome 1
ARL13B	ENST00000471138_3	3	93698999	93772913	NM_18289	CCDS2925	11	Leber	ADP-ribosylation factor-like 13B
C2orf71	ENST00000331664_2	2	29284558	29297127	NM_00102	CCDS42669	2	Leber	chromosome 2 open reading frame 71
C5orf42	ENST00000425232_5	5	37106330	37249530	XM_00524	CCDS34146	52	Leber	chromosome 5 open reading frame 42
C8orf37	ENST00000286688_8	8	96257147	96281429	NM_17796	CCDS6268	6	Leber	chromosome 8 open reading frame 37
CABP4	ENST00000325656_11	11	67222818	67226524	XM_00527	CCDS8166	6	Leber	calcium binding protein 4
CACNA1F	ENST00000376265_X	X	49061523	49089833	NM_00518	CCDS35253	48	Leber	calcium channel, voltage-dependent, L type, alpha 1F subunit
CC2D2A	ENST00000503292_4	4	15471554	15603180	NM_00108	CCDS47026	38	Leber	coiled-coil and C2 domain containing 2A
CEP290	ENST00000552810_12	12	88442797	88535993	XM_00526	CCDS55858	54	Leber	centrosomal protein 290kDa
CEP41	ENST00000223208_7	7	1,3E+08	1,3E+08	XM_00527	CCDS5821	11	Leber	centrosomal protein 41kDa
CNGA3	ENST00000393504_2	2	98962618	99015057	NM_00129	CCDS2034	8	Leber	cyclic nucleotide gated channel alpha 3
CNGB3	ENST00000320005_8	8	87586163	87755903	NM_01909	CCDS6244	18	Leber	cyclic nucleotide gated channel beta 3
CRB1	ENST00000367400_1	1	1,97E+08	1,97E+08	NM_20125	CCDS1390	12	Leber	crumbs homolog 1 (Drosophila)
CRB1	ENST00000535699_1	1	1,97E+08	1,97E+08	NM_00125	CCDS58053	15	Leber	crumbs homolog 1 (Drosophila)
CRX	ENST00000221996_19	19	48325097	48346587	NM_00055	CCDS12706	4	Leber	cone-rod homeobox
CSPP1	ENST00000262210_8	8	67976603	68108498	XM_00525	CCDS43744	29	Leber	centrosome and spindle pole associated protein 1
EYS	ENST00000503581_6	6	64429876	66417118	NM_00114	CCDS47445	43	Leber	eyes shut homolog (Drosophila)
GNAT2	ENST00000351050_1	1	1,1E+08	1,1E+08	NM_00527	CCDS803	8	Leber	guanine nucleotide binding protein (G protein), alpha transducing activity
GPR179	ENST00000342292_17	17	36481493	36499693	NM_00100	CCDS42308	11	Leber	G protein-coupled receptor 179
GRM6	ENST00000231188_5	5	1,78E+08	1,78E+08	NM_00084	CCDS4442	10	Leber	glutamate receptor, metabotropic 6
GUCY2D	ENST00000254854_17	17	7905912	7923657	NM_00018	CCDS11127	20	Leber	guanylate cyclase 2D, membrane (retina-specific)
IFT140	ENST00000426508_16	16	1560428	1662111	XM_00525	CCDS10439	31	Leber	intraflagellar transport 140 homolog (Chlamydomonas)
IMPDH1	ENST00000338791_7	7	1,28E+08	1,28E+08	XM_00525	CCDS34749	17	Leber	IMP (inosine 5'-monophosphate) dehydrogenase 1
INPP5E	ENST00000371712_9	9	1,39E+08	1,39E+08	XM_00526	CCDS7000	10	Leber	inositol polyphosphate-5-phosphatase, 72 kDa
IQCB1	ENST00000310864_3	3	1,21E+08	1,22E+08	XM_00524	CCDS33837	15	Leber	IQ motif containing B1
KCNJ13	ENST00000233826_2	2	2,34E+08	2,34E+08	NM_00224	CCDS2498	3	Leber	potassium inwardly-rectifying channel, subfamily J, member 13
KCNV2	ENST00000382082_9	9	2717502	2730037	NM_13349	CCDS6447	2	Leber	potassium channel, subfamily V, member 2
KIF7	ENST00000394412_15	15	90171208	90198682	XM_00525	CCDS32325	19	Leber	kinesin family member 7
LCA5	ENST00000392959_6	6	80194708	80247147	NM_18171	CCDS4990	9	Leber	Leber congenital amaurosis 5
LRAT	ENST00000336356_4	4	1,56E+08	1,56E+08	NM_00474	CCDS3789	3	Leber	lecithin retinol acyltransferase (phosphatidylcholine--retinol O-acyltransf
MERTK	ENST00000421804_2	2	1,13E+08	1,13E+08	NM_00634	CCDS2094	20	Leber	c-mer proto-oncogene tyrosine kinase
NMNAT1	ENST00000377205_1	1	10003486	10045556	NM_02278	CCDS108	5	Leber	nicotinamide nucleotide adenylyltransferase 1
NPHP1	ENST00000393272_2	2	1,11E+08	1,11E+08	NM_20718	CCDS46385	20	Leber	nephronophthisis 1 (juvenile)
NPHP4	ENST00000378156_1	1	5922878	6052531	XM_00526	CCDS44052	30	Leber	nephronophthisis 4
NYX	ENST00000342595_X	X	41306687	41334963	NM_02256	CCDS14256	2	Leber	nyctalopin
OFD1	ENST00000340096_X	X	13752864	13787472	XM_00527	CCDS14157	23	Leber	oral-facial-digital syndrome 1
PDE6C	ENST00000371447_10	10	95372345	95425767	NM_00620	CCDS7429	22	Leber	phosphodiesterase 6C, cGMP-specific, cone, alpha prime
PDE6G	ENST00000331056_17	17	79617489	79623607	NM_00260	CCDS11783	4	Leber	phosphodiesterase 6G, cGMP-specific, rod, gamma
RBP4	ENST00000371464_10	10	95351444	95360983	NM_00674	CCDS31249	6	Leber	retinol binding protein 4, plasma
RD3	ENST00000367002_1	1	2,12E+08	2,12E+08	NM_00116	CCDS1498	3	Leber	retinal degeneration 3
RDH12	ENST00000551171_14	14	68168603	68201169	NM_15244	CCDS9787	9	Leber	retinol dehydrogenase 12 (all-trans/9-cis/11-cis)
RPE65	ENST00000262340_1	1	68894505	68915642	NM_00032	CCDS643	14	Leber	retinal pigment epithelium-specific protein 65kDa
RPGRIP1	ENST00000400017_14	14	21756136	21819454	XM_00526	CCDS45080	24	Leber	retinitis pigmentosa GTPase regulator interacting protein 1
RPGRIP1L	ENST00000379925_16	16	53634690	53737758	NM_01527	CCDS32447	27	Leber	RPGRIP1-like
SDCCAG8	ENST00000366541_1	1	2,43E+08	2,44E+08	XM_00527	CCDS31075	18	Leber	serologically defined colon cancer antigen 8
SLC24A1	ENST00000261892_15	15	65914270	65948598	XM_00525	CCDS45284	10	Leber	solute carrier family 24 (sodium/potassium/calcium exchanger), membe
SPATA7	ENST00000393545_14	14	88851874	88904800	XM_00526	CCDS9883	12	Leber	spermatogenesis associated 7
TMEM138	ENST00000278826_11	11	61129473	61136981	NM_01646	CCDS8005	5	Leber	transmembrane protein 138
TMEM216	ENST00000334888_11	11	61159832	61166335	XM_005274039		5	Leber	transmembrane protein 216
TMEM237	ENST00000409444_2	2	2,02E+08	2,03E+08	NM_15238	CCDS46490	13	Leber	transmembrane protein 237
TMEM237	ENST00000409883_2	2	2,02E+08	2,03E+08	XM_00524	CCDS46489	13	Leber	transmembrane protein 237
TRPM1	ENST00000397795_15	15	31293264	31393910	NM_00242	CCDS10024	27	Leber	transient receptor potential cation channel, subfamily M, member 1
TRPM1	ENST00000542188_15	15	31293553	31453476	NM_00125	CCDS58347	27	Leber	transient receptor potential cation channel, subfamily M, member 1
TULP1	ENST00000229771_6	6	35465651	35480715	NM_00332	CCDS4807	15	Leber	tubby like protein 1
VPS13B	ENST00000358544_8	8	1E+08	1,01E+08	XM_00525	CCDS6280	62	Leber	vacuolar protein sorting 13 homolog B (yeast)

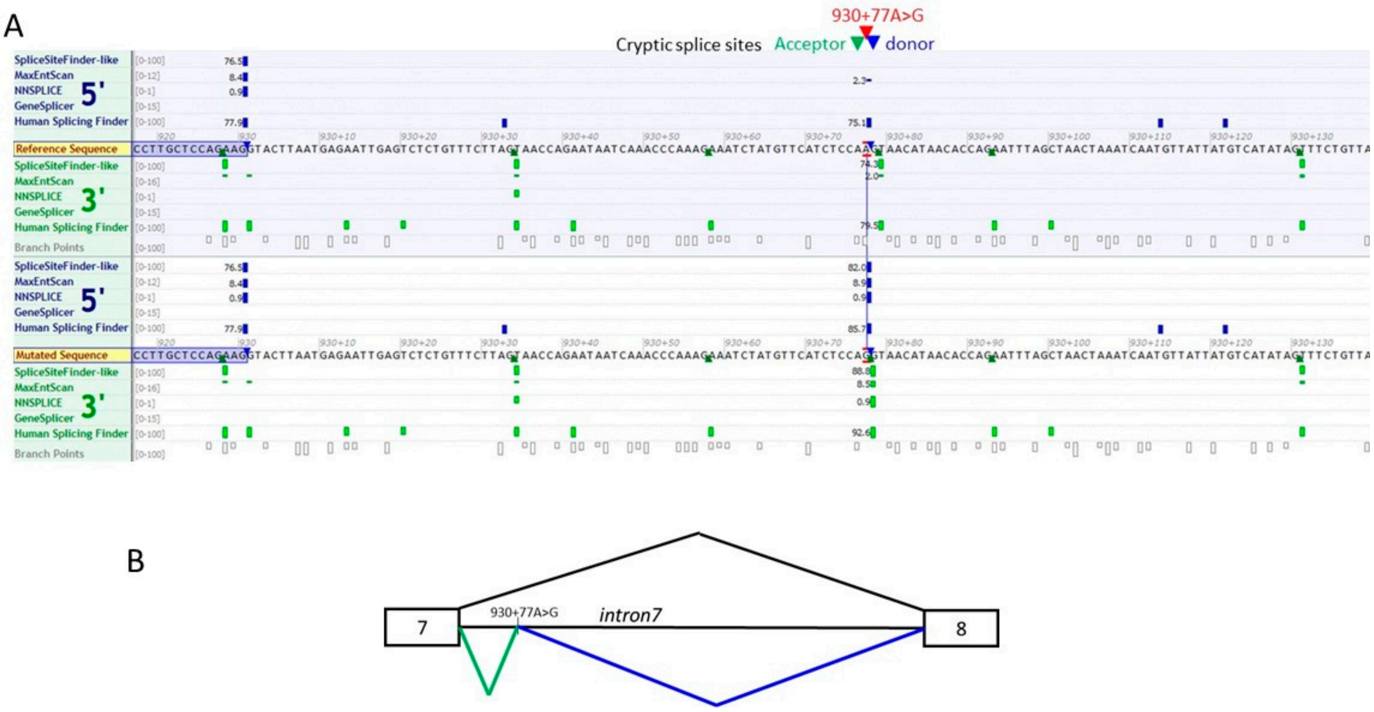

Supplementary Figure S1: Splicing prediction scores around the *RPGRIP1* c.930+77A>G variant in intron 7.
(A) Representation of the wildtype exon-intron 7 junction sequence (Reference Sequence) and the mutant counterpart (c.930+77A>G Mutated sequence) with donor and acceptor splicing sites (blue and green boxes with prediction scores, respectively) according to 5 prediction softwares available through Alamut. Four out of the five softwares predict that the c.930+77A>G variant activates cryptic donor and acceptor splice sites, one nucleotide downstream of the change. These cryptic sites are identified in the wildtype sequence by the Human Splicing Finder and MaxEntScan softwares. The recognition of the cryptic donor site is predicted to lead to the retention of the first 77 nucleotides of intron 7. That of the crytpic acceptor site could result in the retention of the 1139 intronic nucleotides dowstream of the variant.
(B) Schematic representation of exon 7, intron 7 and exon 8 with wildtype (black) and aberrant splicing events that could result from the activation of the cryptic acceptor (green) and donor (blue) splice sites.

Supplementary Figure S2: RT-qPCR analysis of *RPGRIP1* mRNA levels of in retinal, lymphoblasts and fibroblasts from controls and ciliogenesis analysis in control and patient fibroblasts**.**

(A) Analysis of reverse transcribed *RPGRIP1* mRNA extracted from human fetal retina (Retina), control fibroblasts and control lymphoblasts. Relative expression of *RPGRIP1* mRNAs determined by RT-qPCR using *GUSB*, *HPRT1* and *RPLP0* genes as reference. (**B**) Primary cilium and basal bodies staining of fibroblasts from one control and two patients (LCA426 and MON035) using anti-acetylated–tubulin (green) and anti-pericentrin (red) antibodies, respectively. Nuclei are labeled using DAPI (blue). Scale-bar = 10 μm.

(**C**) Quantitative and qualitative analysis of primary cilium biogenesis. The proportions of fibroblasts presenting a primary cilium among cells were calculated by numbering at least 100 cells in at least four fields. Controls corresponds to C1, C2 and C3 pooled values (**D**) Length of cilia axonemes in control and mutant fibroblasts. A minimum of 90 ciliated cells were considered for each cell lines. The Control corresponds to C1, C2 and C3 pooled values. ns: not significant.

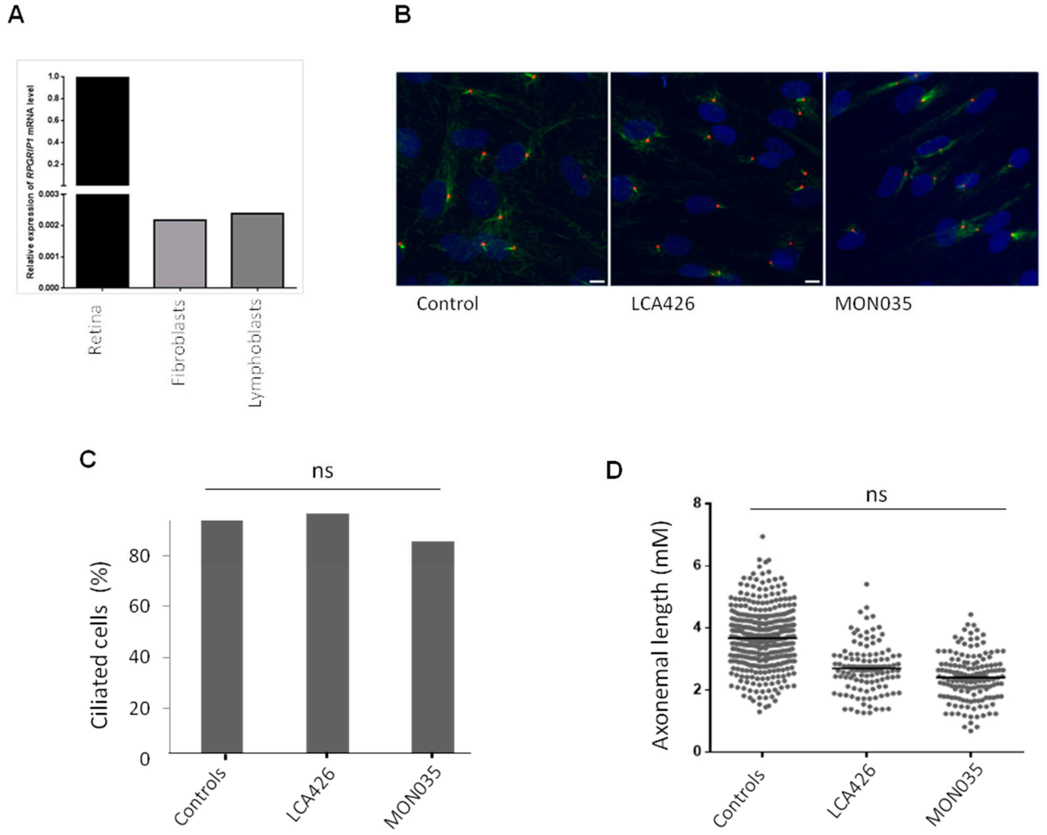

Supplement: Supplementary file 1 [file genes-12-00287-s001.pdf]
